# Cost-effectiveness of a Brief Structured Intervention Program Aimed at Preventing Repeat Suicide Attempts Among Those Who Previously Attempted Suicide: A Secondary Analysis of the ASSIP Randomized Clinical Trial

**DOI:** 10.1001/jamanetworkopen.2018.3680

**Published:** 2018-10-19

**Authors:** A-La Park, Anja Gysin-Maillart, Thomas J. Müller, Aristomenis Exadaktylos, Konrad Michel

**Affiliations:** 1 Personal Social Services Research Unit, Department of Health Policy, London School of Economics and Political Science, London, United Kingdom; 2 Translational Research Centre, University Hospital of Psychiatry, University of Bern, Bern, Switzerland; 3 Private Clinic Meiringen, Meiringen, Switzerland; 4 Department of Emergency Medicine, University Hospital and University of Bern, Bern, Switzerland

## Abstract

**Importance:**

This is the first cost-effectiveness analysis of a brief therapy, the Attempted Suicide Short Intervention Program (ASSIP), for individuals who attempt suicide.

**Objective:**

To explore the cost-effectiveness of the ASSIP intervention in the context of the Swiss health care system.

**Design, Setting, and Participants:**

In this economic evaluation, the cost-effectiveness analysis was performed from a health care perspective between January 2017 and April 2018 using data from a randomized clinical trial conducted between June 2009 and December 2014. Participants were individuals who had attempted suicide and were receiving treatment at a psychiatric university hospital in Switzerland that provides inpatient and outpatient services for suicide attempters referred from an emergency department of a general hospital.

**Interventions:**

The intervention group received 3 manual-based therapy sessions followed by regular personalized letters over 24 months. The control group was offered a single suicide risk assessment.

**Main Outcomes and Measures:**

The main economic analysis explored cost per suicide attempt avoided expressed in 2015 Swiss francs (CHF). Cost-effectiveness planes were plotted and cost-effectiveness acceptability curves calculated.

**Results:**

One hundred twenty participants (mean [SD] age, 37.8 [14.4] years; 66 [55%] women and 54 [45%] men) were assigned to an intervention group or a control group, each with 60 participants. At 24 months of follow-up, 5 suicide attempts were reported in the ASSIP group among 59 participants with follow-up data available, and 41 were reported in the control group among 53 participants with follow-up data available. The ASSIP group had higher intervention costs, with CHF 1323 vs CHF 441 for the control group. At 24 months of follow-up, psychiatric hospital costs were lower in the ASSIP group than in the control group, although this difference was not significant (mean [SD], CHF 20 559 [38 676] vs CHF 45 488 [73 306]; mean difference, CHF −16 081; 95% CI, CHF −34 717 to 1536; *P* = .11). General hospital costs were significantly lower for the ASSIP group. Total health care costs were also lower, but the difference was not significant (mean [SD], CHF 21 302 [38 819] vs 41 287 [74 310]; difference, CHF −12 604; 95% CI, CHF −29 837 to 625; *P* = .14). In a base-case analysis, ASSIP was dominant, with significantly fewer reattempts at lower overall cost. The intervention had a 96% chance of being less costly and more effective. A sensitivity analysis showed a 96% and 95% chance of ASSIP being more effective and less costly at willingness-to-pay levels of CHF 0 and CHF 30 000, respectively.

**Conclusions and Relevance:**

The ASSIP intervention is a cost-saving treatment for individuals who attempt suicide. The findings support the use of ASSIP as a treatment for suicide attempters. Further studies are needed to determine cost-effectiveness in other contexts.

**Trial Registration:**

ClinicalTrials.gov Identifier: NCT02505373.

## Key Points

**Question:**

Is the Attempted Suicide Short Intervention Program, a brief novel therapy for individuals who attempt suicide, cost-effective compared with a suicide risk assessment?

**Findings:**

In this economic evaluation conducted alongside a randomized clinical trial that included 120 Swiss adults, the intervention group had significantly lower levels of suicide reattempts compared with the control group at lower health care costs, with a 96% chance of being less costly and more effective in a sensitivity analysis.

**Meaning:**

In the context of the Swiss health care system, the Attempted Suicide Short Intervention Program was found to be cost saving as a follow-up treatment for attempted suicide.

## Teaser

This secondary analysis of the ASSIP randomized clinical trial compares the cost-effectiveness of a brief novel therapy vs usual care for preventing repeat suicide attempts among patients in Switzerland who had previously attempted suicide.

## Introduction

According to World Health Organization statistics, more than 800 000 people die of suicide every year, and 10 to 20 times more people attempt suicide.^1^ Attempted suicide is one of the most frequent reasons for emergency hospital admissions.^2^ In the United States, it is estimated that each year 1.4 million people attempt suicide.^3^ Between 14% and 22% of these individuals will have repeat suicide attempts within a year.^4^ Furthermore, people with a history of attempted suicide have up to 100 times increased risk of completed suicide compared with the general population.^5^

The economic costs associated with health and social care services due to suicidal behaviors, such as medical emergency treatment, general and psychiatric hospitalizations, outpatient care, informal care costs, and productivity losses, are enormous. For example, in the United States, the yearly costs of suicidal behavior, which includes suicide and attempted suicide, have been estimated at $93.5 billion.^6^ In Scotland, the costs of all suicides per year have been reported to be over £1 billion.^7^ For attempted suicide, the mean costs at a hospital in Oxford in the United Kingdom, including psychosocial assessment and general hospital services, were estimated to be £809 per patient. The extrapolated costs for England were £162 million per year.^8^ In Switzerland, the total medical and psychiatric treatment costs of suicide attempts for a population of 8 million people were estimated at CHF 191 million per year.^9^

The evidence that specific treatments are effective in reducing the risk of repeated attempts so far is limited.^10,11,12,13^ Economic evaluations of effective interventions to prevent suicidal behaviors are scarce.^14^

The ASSIP intervention is a novel therapy for people who attempt suicide. It is brief, highly structured, and relatively easy to learn and to implement. In a randomized clinical trial (RCT) with a 24-month follow-up, ASSIP was associated with an 80% reduction in the risk that patients would reattempt suicide.^15^ As part of this trial, an economic evaluation was conducted post hoc to explore the cost-effectiveness of ASSIP to inform health system resource allocation decisions.

## Methods

### Trial Design and Participants

Patients who had attempted suicide and who had presented to the emergency department at the general hospital at the University of Bern in Switzerland were recruited from inpatient or outpatient follow-up treatment and were randomly assigned to either the ASSIP or control groups from June 2009 until December 2012 with a 2-year follow-up ending in December 2014. This economic evaluation is reported here as recommended in the Consolidated Health Economic Evaluation Reporting Standards (CHEERS) reporting guideline.^16^ The trial protocol is available in Supplement 1. The cost-effectiveness analysis was subsequently conducted between January 2017 and April 2018. The term *attempted suicide* used in the study followed the definition by Silverman et al as a “self-inflicted, potentially injurious behavior with a nonfatal outcome for which there is evidence (either explicit or implicit) of intent to die.”^17^

### Randomization and Masking

Patients who gave informed consent were randomly assigned using shuffled unmarked sealed envelopes between groups. Suicidality was assessed with the Suicide Status Form and individuals were diagnosed using the *International Classification of Diseases, Tenth Revision*.^18^ The study was approved by the local research ethics committee of the University of Bern in accordance with the Declaration of Helsinki.^19^

### Interventions

The ASSIP intervention consisted of three 60-minute face-to-face sessions, followed by semistructured personalized letters sent to individuals 6 times over 24 months.^20^ The conceptual model for the intervention considers suicidal behavior as a goal-oriented action rather than a form of illness.

The ASSIP protocol is described in detail in the manual.^20^ In the first session, the therapist focuses on the patient’s narrative of the background of the suicidal crisis and establishes a collaborative working alliance. In a video-playback session, patient and therapist collaboratively review the recorded interview. A psychoeducational text with the title “Suicide Is Not a Rational Act” is handed out to the patient. In the third session, a case conceptualization is collaboratively formulated, identifying the patient’s personal vulnerability and suicide trigger, warning signs, and safety strategies tailored to individual needs. Patients are reminded of their own personal safety strategies with a credit-card-sized summary listing emergency contact details. In the control group of the RCT, individuals received 1 single session for suicide risk assessment, which was sent to the patient’s physician.

### Clinical Outcomes

Outcomes were measured at 6, 12, 18, and 24 months after the intervention. The primary outcome measure was the total number of suicide attempts in both groups. For further details, see the original publication.^15^

### Resource Use and Costs

The economic evaluation was performed from a health care system perspective. In Switzerland this is of interest not only to hospitals, but also to insurance providers and the cantonal (regional) health authorities who are the major funders of health care services. Resource use data on the number of inpatient days and the number of outpatient sessions attended were collected from patient self-reports every 6 months. Costs of resources used were estimated by applying relevant unit costs in Swiss Francs (CHF) for the financial year 2015. General hospital emergency department hospitalizations related to suicidal behaviors were estimated retrospectively by examining patient hospital records. Training costs were added in a sensitivity analysis to explore the implications for future implementation costs. This seemed relevant because the 2 health care professionals providing ASSIP in this study did not need training as developers of the treatment protocol. Unit costs and training costs obtained from university hospital staff are presented in eTable 1 and eTable 2 in Supplement 2. Alternative scenarios explored whether results were robust by excluding intervention costs, using undiscounted costs and effects, and including imputed values.

### Statistical Analysis

The ASSIP trial was powered using the results of previous trials.^15^ Economic analyses were performed based on a modified intention-to-treat principle^21^ by including all study participants who had any data for at least 1 follow-up point available during the entire 24 months in the group to which they had initially been randomized in the main analysis.

Costs were compared at each point over the entire 24-month period. They are reported as mean values with standard deviations. Baseline costs included costs for previous suicide attempts related to days in the hospital as well as outpatient costs incurred in the 6 months prior to intervention. It seemed important to adjust mean costs for these baseline costs due to a significant difference reported in the original study,^15^ especially in outpatient sessions. Mean differences and 95% confidence intervals were obtained by bias-corrected and accelerated nonparametric bootstrap regressions^22^ using 1000 replications that included baseline costs as a covariate.

In our study, cost-effectiveness was explored as the incremental cost to prevent 1 additional attempted suicide using the net-benefit approach.^23^ In the base case analysis, costs and effects beyond 12 months were discounted at 3.5% and 1.5%, respectively, as the intervention lasted over 1 year.^24^

Although there is no officially recommended discount rate in Switzerland at a national level, discounting rates in health economic evaluations by convention generally range from 1.5% to 5% to reflect the time preference correctly.^25^ It is commonly recommended to use lower discounting rates for the effectiveness of public health interventions as well as preventive interventions such as suicide or attempted suicide prevention strategies.^26^ This is because the benefits from a preventive intervention are more likely to be realized over a longer time horizon. If the same discount rates are used for both costs and effects, the potential benefits, which would be realized in the longer term, can be undervalued. Therefore, to prevent this and reflect reality, we applied the lower discount rate of 1.5% for effects and 3.5% for costs, as recommended by the National Institute for Health and Care Excellence in the United Kingdom.^26^ In addition, we varied discount rates from 0% (undiscounted) to 6% for both costs and effects to test the sensitivity of the results by using higher discount rates as a conservative estimate.

A joint distribution of incremental mean costs and effects between the 2 groups was generated using nonparametric bootstrapping. These data were used to explore the probability that the intervention is the optimal choice, subject to a range of possible threshold values that a decision maker might be willing to pay for an improvement in our outcome of interest in a Swiss context. Cost-effectiveness acceptability curves were generated by plotting these probabilities for a range of plausible threshold values. Missing costs and outcomes were imputed using multiple imputation chained equations with predictive mean matching to deal with skewness. All analyses were 2-tailed and a *P* value of less than .05 was considered significant. The data were analyzed using SPSS statistical software version 21 (IBM) and STATA statistical software version 15 (StataCorp).

## Results

### Effectiveness

One hundred twenty participants (mean [SD] age, 37.8 [14.4] years; 66 [55%] women and 54 [45%] men) were included in the study with 60 in each group. No significant differences were found in study participants’ characteristics between the 2 groups, except in the number of outpatient visits before the suicide attempt (median [IQR] number of visits, 8 [2.25-15.75] in the intervention vs 2.5 [0-10.5] in the control group).^25^ The CONSORT diagram for the trial and baseline characteristics of the participants are available in the previously published article.^15^

At 24 months of follow-up, 5 suicide attempts were recorded of 59 participants in the ASSIP group for whom follow-up data were available, compared with 41 attempts of 53 participants in the control group. The mean (SD) number of suicide attempts per person at 2 years of follow-up was 0.084 (0.278) in the ASSIP group and 0.769 (1.833) in the control group (incremental mean [SE] difference, −0.685 [0.263]; 95% CI, −1.201 to −0.169; *P* = .009).

### Intervention Costs

Three sessions were provided in the ASSIP group and 1 session in the control group. Sessions normally consisted of 60 minutes face to face with patients plus 30 minutes for record keeping and administrative work. Attendance rates were 100% in each group. The mix of staff time to deliver the interventions to service users was approximately 40% by a psychiatrist and 60% by a clinical psychologist. The ASSIP group had higher intervention costs, with CHF 1323 vs CHF 441 for the control group. Because of the different number of sessions provided, the intervention costs were 3 times higher in the ASSIP group compared with the control group.

### Resource Use for Psychiatric Care Services

There were 1176 and 2915 psychiatric inpatient days in total in the ASSIP group and the control group, respectively, and 2041 and 1638 outpatient sessions in the 2 groups, respectively. On average, patients in the ASSIP group had fewer days of inpatient care than the control group (mean [SD], 21 [57] days vs 60 [111] days). On the other hand, ASSIP participants had a slightly higher mean number of outpatient sessions compared with patients in the control group (mean [SD], 36 [44] sessions vs 33 [44] sessions). This suggests a shift of costs from more expensive forms of inpatient care to less costly outpatient care.

### Health Service Costs

Mean costs for inpatient care services in the psychiatric hospital, measured every 6 months, were higher for people in the control group than those in the ASSIP group, although there was no clear pattern in outpatient costs between the 2 groups. However, no significant differences in psychiatric hospital costs were found when we looked at the group difference in psychiatric service costs as snapshots at different time points, as shown in Table 1.

**Table 1.** Mean Psychiatric Health Care Costs for Every 6-Month Period at 6, 12, 18, and 24 Months

Types of Costs and Time Points	Mean (SD)		Unadjusted Cost Difference, Mean	Adjusted Cost Difference, Mean (95% CI)	*P* Value
	ASSIP Cost, CHF	Control Cost, CHF			
**Inpatient Care**					
Baseline (n = 57 vs 59)	62 248 (156 605)	69 871 (161 211)	−7623	−7622 (−60 963 to 47 570)	.81
0-6 mo (n = 42 vs 42)	9743 (19 636)	22 267 (35 977)	−12 524	−9805 (−21 553 to 677)	.11
6-12 mo (n = 37 vs 36)	4031 (17 326)	12 137 (26 444)	−8106	−6037 (−14 427 to 844)	.24
12-18 mo (n = 36 vs 33)	4092 (14 325)	5816 (18 685)	−1724	−1725 (−9984 to 5416)	.69
18-24 mo (n = 54 vs 38)	1169 (4097)	8978 (29 664)	−7809	−7809 (−19 181 to 814)	.19
**Outpatient Care**					
Baseline (n = 58 vs 60)	2176 (2338)	1556 (2622)	620	620 (−247 to 1444)	.17
0-6 mo (n = 43 vs 42)	3676 (3700)	2514 (3163)	1162	672 (−512 to 1831)	.29
6-12 mo (n = 37 vs 36)	2133 (2612)	2493 (4076)	−360	−647 (−2418 to 499)	.52
12-18 mo (n = 36 vs 35)	2258 (3165)	1576 (2331)	682	682 (−540 to 1946)	.31
18-24 mo (n = 54 vs 38)	1570 (2150)	1925 (3344)	−355	−355 (−1716 to 838)	.58

Abbreviations: ASSIP, Attempted Suicide Short Intervention Program; CHF, Swiss francs.

From a health care payer perspective, psychiatric hospital costs, including inpatient and outpatient care costs, were lower in the intervention group compared with the control group. This difference was not significant (mean [SD], CHF 20 559 [38 676] vs CHF 45 488 [73 306]; adjusted mean difference, CHF −16 081; 95% CI, CHF −34 717 to 1536; *P* = .11). General hospital costs were significantly lower in the intervention group than the control group (mean [SD], CHF 456 [1511] vs CHF 4186 [9966]; adjusted mean difference, CHF −3045; 95% CI, −6333 to −1128; *P* = .005). General hospital costs included assessment, consultation, and liaison psychiatric services. Overall, total health care costs were lower in the ASSIP group than the control group. This difference was not significant (mean [SD], CHF 21 302 [38 819] vs CHF 41 287 [74 310]; difference, CHF −12 604; 95% CI, CHF −29 837 to 625; *P* = .14), as shown in Table 2.

**Table 2.** Mean Total Costs Per Participant Over 24-Month Follow-Up

Cost Type	Mean (SD)		Unadjusted Mean Cost Difference	Adjusted Mean Cost Difference (95% CI)	*P* Value
	ASSIP Cost, CHF	Control Cost, CHF			
Intervention (n = 60 vs 60)	1323 (0)	441 (0)	882	882 (882 to 882)	.002
Psychiatric hospital (n = 54 vs 42)	20 559 (38 676)	45 488 (73 306)	−24 929	−16 081 (−34 717 to 1536)	.11
General hospital (n = 59 vs 53)	456 (1511)	4186 (9966)	−3730	−3045 (−6333 to −1128)	.005
Total costs including intervention costs (n = 60 vs 60)	21 302 (38 819)	41 287 (74 310)	−19 985	−12 604 (−29 837 to 625)	.14

Abbreviations: ASSIP, Attempted Suicide Short Intervention Program; CHF, Swiss francs.

Regarding the incremental cost-effectiveness ratio, the intervention group had both a much lower level of recurring attempted suicides (mean [SD] attempts per person, 0.084 [0.278] vs 0.769 [1.833]; mean difference, −0.685; 95% CI, −1.201 to −0.169; *P* = .009) and lower costs (mean [SD] costs per person, CHF 21 302 [38 819] vs CHF 41 287 [74 310]; mean difference, −12 604; 95% CI, −29 837 to 625; *P* = .14) than the control group. In this case, ASSIP is considered by health economists to be the dominant intervention. The intervention had a 96% chance of being less costly and more effective. By convention, the incremental cost-effectiveness ratio value is not reported. The analysis strongly suggests that ASSIP would be a cost-saving strategy.

In a series of sensitivity analyses, we explored the impacts of varying assumptions by decreasing costs (ie, excluding intervention costs from total costs in the main analysis) and by increasing costs (ie, adding training costs) to see whether changes in cost components would make any difference to the final conclusions. In addition, discount rates were varied between 0% (undiscounted) to the most conservative estimates of 6% for both costs and outcomes. Sensitivity analyses had no impact on the interpretation of the findings, consistently showing lower costs in the ASSIP group than in the control group. Table 3 shows that these differences in costs were not significant.

**Table 3.** Sensitivity Analyses for 24-Month Costs Per Person

Assumptions and Cost Scenarios	Mean (SD)		Unadjusted Mean Difference	Adjusted Mean Difference (95% CI)	*P* Value
	ASSIP Cost, CHF	Control Cost, CHF			
Excluding intervention costs	20 318 (39 063)	46 240 (77 531)	−25 922	−17 031 (−35 014 to 952)	.06
Including training costs	21 515 (38 819)	41 287 (74 310)	−19 772	−12 451 (−29 875 to 1617)	.16
Undiscounted costs	21 531 (39 257)	41 725 (75 368)	−20 194	−12 737 (−29 291 to 1427)	.14
Imputed costs	21 302 (38 819)	41 287 (74 310)	−19 985	−13 851 (−30 681 to 2979)	.11
Costs discounted at 6%	21 148 (38 526)	40 991 (73 601)	−19 843	−12 615 (−31 842 to 1597)	.17

Abbreviations: ASSIP, Attempted Suicide Short Intervention Program; CHF, Swiss francs.

**Figure 1.**
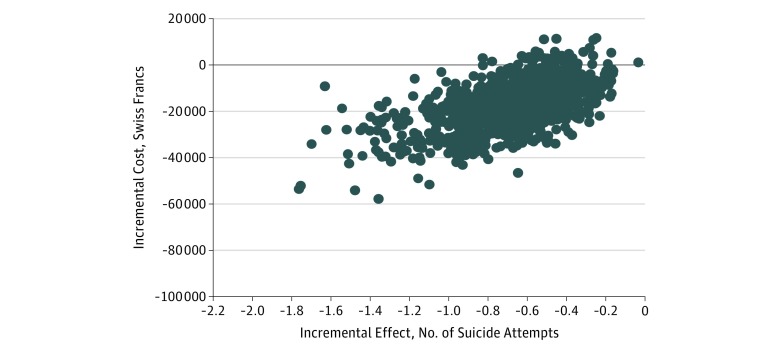
Cost-effectiveness Plane for 24-Month Results for the Number of Suicide Attempts The majority of bootstrapped paired dots representing differences in pairs of costs and effects were scattered across the southwest quadrant. This indicates that the Attempted Suicide Short Intervention Program is highly likely to be less costly and more effective in reducing the number of reattempts, compared with the control group. The x-axis represents the incremental effect in terms of the number of suicide attempts. The lower the number, the better the effect.

This is also confirmed in the cost-effectiveness plane shown in Figure 1. In the scatterplot, the majority of bootstrapped paired dots representing differences in pairs of costs and effects were scattered across the southwest quadrant. This indicates that the ASSIP intervention is highly likely to be less costly and more effective in reducing the number of reattempts compared with the control group (negative incremental effect is desirable, as this means reduced attempted suicides).

**Figure 2.**
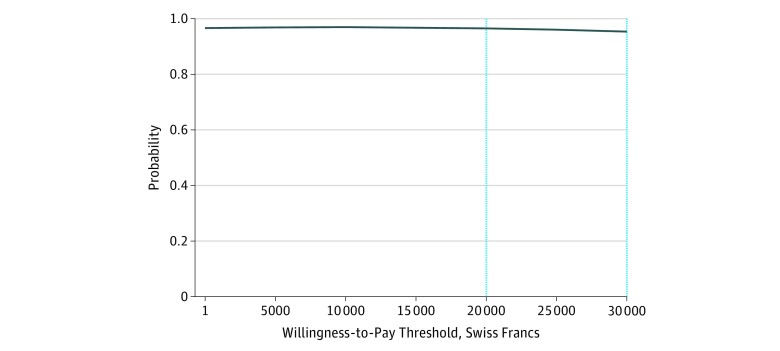
Cost-effectiveness Acceptability Curve The graph shows the probability of the Attempted Suicide Short Intervention Program being cost-effective at different willingness-to-pay levels.

In Figure 2, the cost-effectiveness acceptability curve shows that ASSIP would potentially be cost saving even at the lower ranges of willingness to pay. There is a 96% chance of ASSIP being less costly and more effective if willingness-to-pay is CHF 0 and a 95% chance at CHF 30 000. Therefore, it can be concluded that the sensitivity analyses show highly stable results and that ASSIP is a worthwhile investment.

## Discussion

The probability that ASSIP was cost saving at 24 months was very high even at very low levels of willingness to pay. Most of the cost savings were driven by significantly lower costs for general hospital services and less use of inpatient care services in the ASSIP group. Perhaps the absence of a significant difference in psychiatric health service costs, despite the consistently lower inpatient costs in the intervention group over time, could be attributed to the lack of power to detect significant differences at each time point due to the small sample size. In theory, it is also partly because larger sample sizes are generally required to detect a significant difference in costs than in effects, although in reality, power calculations for RCTs are usually based on primary clinical outcomes rather than costs.^23^ Therefore, more studies with larger sample sizes might be helpful to confirm the significant difference in costs in future RCTs. However, this is indeed an ethical dilemma in RCTs with suicidal patients. To replicate an RCT using ASSIP in different health care systems, the control condition must always include an adequate treatment as usual, which includes the possibility of emergency contacts during follow-up.

Establishing the cost-effectiveness of new treatments should have a high priority, given the number of suicide attempts and the burden on health care systems.^2^ For instance, only half of individuals who attempt suicide and are discharged from emergency departments received follow-up outpatient care for their mental health within 1 month in the United States.^27^ The ASSIP intervention can be recommended for patients discharged from psychiatric or general hospital care, given the fact that the months after hospital discharge are associated with a particularly high suicide risk.^28^ Because the program manual is brief and easy to understand for the service users, the potential dropout rate in clinical practice is expected to be low.

### Transferability of Findings

This study was conducted in Switzerland, a country with a relatively high-quality medical care system. Psychiatric care for suicidal patients is provided as a consultation service to medical emergency departments and as inpatient and outpatient treatment in psychiatric institutions. In addition, Switzerland has a high number of psychiatrists in private practice, offering psychiatric and psychotherapeutic treatment. The funding of health care institutions is partly provided by the state and partly by the mandatory sickness insurance. Although Switzerland does not have a national health care system, the provision of care to suicidal patients is generally comparable to most other Western countries. An earlier study from Switzerland^9^ reported average costs of €16 700 for psychiatric inpatient treatment for attempted suicide, while in Australia,^13^ hospital costs between A$3015 and A$8534 were reported. The same authors suggested that by reducing the suicide repetition rate from 16% to 13% in Australia, cost savings of A$3 041 166 could be achieved. Considering that the ASSIP trial found a reduction from 27% to 8% in patients reattempting suicide within 24 months, the potential savings in national health care expenditures could be substantial.

Length of psychiatric inpatient care after attempted suicide can vary between institutions and health care systems. In our study, the average length of psychiatric inpatient care was 21 days for the ASSIP group and 60 days for the control group. There is a lack of internationally comparable data on the length of hospitalizations for suicidal patients. A US analysis reported a mean length of stay in the emergency department of 5 days.^29^ More studies are needed to confirm generalizability issues between countries and to improve comparability at an international level.

However, with the majority of worldwide suicides occurring in low- and middle-income countries,^1^ there is an urgent need for replication studies in different national and sociocultural settings. This would require dissemination of ASSIP to different cultural settings and health care systems.

### Strengths

To our knowledge, our study is the first cost-effectiveness analysis for suicide attempt prevention alongside an RCT in Switzerland, although there have been a few studies for economic evaluations of general self-harm prevention. Given the scarcity of economic evidence on effective attempted suicide prevention strategies in the world, our study will contribute to filling the research gap in the field and help relevant stakeholders, including funders, commissioners, and health care professionals, to make rational resource allocation decisions.

The ASSIP intervention is brief, simple, and potentially a cost-saving program. In the base case analysis, ASSIP was the dominant strategy. The results were robust in a series of sensitivity analyses. The intervention is relatively easy to learn, and the training curriculum, including case supervision, amounts to a total of 4 to 5 days per therapist trained. Therapists using ASSIP are expected to have a background of basic psychotherapy training and practical experience in dealing with suicidal patients. Implementation of effective treatments in some countries may not be feasible owing to treatment costs and the lack of availability of trained health professionals.^30^ To help build capacity, a train-the-trainer schema is under development.

The integration of ASSIP into regional and national health care would depend on various health system infrastructures. For instance, in Switzerland, psychiatric outpatient treatment is readily available and covered by the mandatory insurance system, while in many other health care systems, those who attempt suicide can only be given minimal follow-up treatments or none at all. In Bern, the special ASSIP clinic was part of the university psychiatric service, where it was feasible to provide ASSIP as an add-on treatment to inpatients or outpatients alongside the patient care pathway, leading to earlier discharge from inpatient care.

### Limitations

No officially recommended willingness-to-pay levels exist in terms of cost per self-harm event averted. Therefore, we recommend that future studies explore further economic impacts using generic outcome measures, such as data on quality of life. Cost-utility analyses would facilitate international comparisons and might help decision makers to optimize resource allocation. In addition, if a broader societal perspective was adopted, including impacts on productivity gained through effective intervention, the potential for the cost savings would be even greater. Given a significant association found between deliberate self-harm and violent crime,^31^ the potential benefits and cost-savings would be even greater if we take a broader perspective beyond the health sector.

## Conclusions

This study found ASSIP to be cost saving in reducing the number of suicide attempts in the study population. The results suggest that ASSIP can be considered for scale-up in various local and national settings. Health care professionals will be interested in replicating studies in different regional and national health care systems. In the future, more economic evaluation studies with larger sample sizes might be useful to help determine the cost-effectiveness of ASSIP in different settings at an international level. However, it should be noted that treatments for attempted suicide and the related costs depend on the local, regional, and national mental health care systems and cultural contexts.

## Notes

Supplement 1. **Trial Protocol**

Supplement 2. **eTable 1.** Unit Costs **eTable 2.** Breakdown of Total Training Costs for the ASSIP

Supplement 3. **Data Sharing Statement**
