# Supplementary material for: Cost-effectiveness of a Brief Structured Intervention Program Aimed at Preventing Repeat Suicide Attempts Among Those Who Previously Attempted Suicide: A Secondary Analysis of the ASSIP Randomized Clinical Trial
Source: JAMA Netw Open. 2018 Oct 19;1(6):e183680. doi: 10.1001/jamanetworkopen.2018.3680 (PMC6324444; doi:10.1001/jamanetworkopen.2018.3680)
Supplement: Supplement 2. — eTable 1. Unit Costs eTable 2. Breakdown of Total Training Costs for the ASSIP [file jamanetwopen-1-e183680-s002.pdf]

Supplementary Online Content

Park A-L, Gysin-Maillart A, Müller TJ, Exadaktylos A, Michel K. Cost-effectiveness of a brief structured intervention program aimed at preventing repeat suicide attempts among those who previously attempted suicide: a secondary analysis of the ASSIP randomized clinical trial. *JAMA Netw Open*. 2018;1(6):e183680. doi:10.1001/jamanetworkopen.2018.3680

eTable 1. Unit Costs

eTable 2. Breakdown of Total Training Costs for the ASSIP

This supplementary material has been provided by the authors to give readers additional information about their work.

© 2018 Park A-L et al. *JAMA Network Open.*

eTable 1: Unit costs

**Cost components**	**Unit**	**Unit cost (CHF, Swiss Francs)**
Intervention		
Psychiatrist	60 minutes	CHF 369.86
Clinical psychologist	60 minutes	CHF 256.00
Health services		
Psychiatric inpatient care	Per day	CHF 660
Outpatient care by psychiatrist in private practice	60-minutes	CHF 200.33
General hospital care service	Per case	CHF 5488

source: communications with hospital staff at the University hospital. Abbreviations: ASSIP, Attempted Suicide Short Intervention Program; CHF, Swiss Francs.

© 2018 Park A-L et al. *JAMA Network Open.*

eTable 2: Breakdown of total training costs for the ASSIP

**1.Preparation for and running training sessions**	**Cost (CHF, in 2015)**
i.Basic training course:	993
ii. Case supervision: 3 cases individual supervision	993
iii. Follow-up: 2 days follow-up meeting	662
Total cost	2648
**2.Trainees' time to attend training**	
i.Basic training: 3 days x 8 hours=24 hours	4808
ii. Case supervision: 3 Skype-based supervisions x1 .5 hours= 4.5 hours	902
iii. Follow-up: 2 days x 8 hours=16 hours	3205
Total cost: psychiatrist hourly rate (200.33) x 44.5 hours	8915
**3.Travel and expenses for clinical psychologist to attend training**	
i. Return flight tickets in Europe	453
ii. 3-night accommodation	745
Total cost	1198
**Grand total**	**12761**

Abbreviations: ASSIP, Attempted Suicide Short Intervention Program; CHF, Swiss Francs.

© 2018 Park A-L et al. *JAMA Network Open.*
