# Supplementary material for: Cost-effectiveness of a Brief Structured Intervention Program Aimed at Preventing Repeat Suicide Attempts Among Those Who Previously Attempted Suicide: A Secondary Analysis of the ASSIP Randomized Clinical Trial
Source: JAMA Netw Open. 2018 Oct 19;1(6):e183680. doi: 10.1001/jamanetworkopen.2018.3680 (PMC6324444; doi:10.1001/jamanetworkopen.2018.3680)
Supplement: Supplement 3. — Data Sharing Statement [file jamanetwopen-1-e183680-s003.pdf]

Data Sharing Statement

Park. Cost-effectiveness of a Brief Structured Intervention Program Aimed at Preventing Repeat Suicide Attempts Among Those Who Previously Attempted Suicide. *JAMA Network Open*. Published October 19, 2018. 10.1001/jamanetworkopen.2018.3680

Data
Data available: No
